# Mechanistic Study and Regulatory Effects of Chloride Ions on the B-Z Oscillating Reaction

**DOI:** 10.3390/molecules30153210

**Published:** 2025-07-31

**Authors:** Lidan Niu, Lijuan Zhou, Qihui Wang, Wenjing Yang

**Affiliations:** 1 Key Laboratory of Condiment Supervision Technology, State Administration for Market Regulation, Chongqing Institute for Food and Drug Control, Chongqing 400713, China; 18298237411@139.com; 2 School of Chemistry and Chemical Engineering, Chongqing University, Chongqing 400044, China; 202318021066@stu.cqu.edu.cn; 3 School of Mechanical and Intelligent Manufacturing, Chongqing University of Science and Technology, Chongqing 401331, China; qihuiwang@cqust.edu.cn

**Keywords:** B-Z reaction, Cl^−^, mechanism, induction time, multivariate analysis

## Abstract

This work investigated the mechanistic role of chloride ions (Cl^−^) in the Belousov–Zhabotinsky (B-Z) oscillating reaction. We conducted a multivariate statistical analysis of the B-Z response, established a quadratic polynomial regression model, and determined the contributions of the experimental parameters to the induction time. The results indicate that the relationship between the experimental parameters and the induction time is often nonmonotonic, exhibiting secondary dependence. Then, we studied the influence mechanism by which Cl^−^ affects the B-Z reaction system. Both experimental and theoretical studies indicate that as the concentration of Cl^−^ increases, the system becomes more active as the activation energy increases. When the Cl^−^ concentration is less than 1 mmol/L, the induced apparent activation energy remains relatively constant. However, as the Cl^−^ concentration increases from 1.00 mmol/L to 2.00 mmol/L, the induced apparent activation energy increases rapidly from 50 kJ/mol to 120 kJ/mol, which severely hinders the induction period and then increases the induction time.

## 1. Introduction

Nonlinear phenomena play an essential role in various fields of natural sciences and engineering [1,2,3,4,5]. In particular, nonlinear chemical reactions are observed in various domains [6,7,8,9,10,11,12,13,14,15]. The classical Belousov–Zhabotinsky (B-Z) oscillating reaction refers to the oscillatory oxidation of citric acid catalyzed by cerium [16]. The reaction is typically carried out either as a well-stirred homogeneous system or a spatially distributed reaction–diffusion system [17], producing spatiotemporal rhythms and patterns [18]. The B-Z mixture is an acidic solution containing bromate ions and an oxidizable organic substrate [malonic acid (MA)] that causes the concentration of certain reaction intermediates to oscillate in a suitable single-electron REDOX catalyst, typically cerium (IV) or manganese (II) salts or iron present in proteins]. Proton catalysis plays a role in the electron transfer reaction involving the [Fe(phen)_3_]^3+^ species. This reaction involves a fairly complex mechanism [19,20,21], the main features of which were described in the minimal FKN model by Field, Körös, and Noyes in 1972 and further elaborated in the Gyorgyi–Turanyi–Field (GTF) model [22]. The presence of chloride ions (Cl^−^) in the oscillating system disrupts the pulse signal and alters the system’s induction time parameters. However, there are no relevant reports on the mechanism by which the Cl^−^ affects the B-Z reaction. Therefore, research on how chloride affects the B-Z oscillation reaction is crucial for advancing the understanding of this system.

The concentration of Cl^−^ in natural water, industrial wastewater, and domestic sewage is one of the criteria for evaluating water quality. Existing methods for chloride ion detection mainly include titration, spectrophotometry, and ion chromatography. Titration is operationally simple but lacks precision and has limited detection limits. Spectrophotometry is highly sensitive, uses simple instrumentation, is rapid, and is widely applied, although its accuracy is relatively low. Ion chromatography offers high accuracy but is time-consuming. An electrochemical system based on the B-Z oscillating reaction can generate oscillating potential pulse signals. The addition of Cl^−^ to the oscillating system interferes with these pulse signals, causing changes in the induction time parameter. By analyzing the chemical information reflected by the changes in induction time, chloride ion concentrations can be detected. To date, there have been no reports on the application of B-Z oscillating reactions for chloride ion detection. Therefore, conducting research on the detection of Cl^−^ via the B-Z oscillating reaction and performing kinetic analysis holds significant importance and application value.

In this work, we optimized the process conditions of the sorium-catalyzed bromic acid-malonic acid B-Z oscillation reaction system. The effects of experimental parameters such as KBrO_3_, CH_2_(COOH)_2_, H_2_SO_4_, (NH_4_)_4_Ce(SO_4_)_4_, and temperature on the potential pulse oscillation signal were studied, and a quadratic polynomial regression model was established. The contributions of these parameters to changes in induction time were determined. Additionally, the correlation between the induction time and Cl^−^ concentration was studied, and its kinetics were analyzed to explain its underlying influence mechanism. The main research results are as follows:

1. The process conditions of the B-Z oscillatory response system were optimized. The research results show that at 30 °C, with the H_2_SO_4_ concentration is 3.00 mol/L, the CH_2_(COOH)_2_ concentration is 1.10 mol/L, the concentration of (NH_4_)_4_Ce(SO_4_)_4_ is 0.007 mol/L, and the KBrO_3_ concentration is 0.30 mol/L, which are the best process conditions for the oscillation response system. The potential oscillation pulse signal generated by this system has excellent stability, reproducibility, and periodicity. The induction time is 136.4 s, and the oscillation period is 39.0 s.
2. By studying the influence of each component concentration and temperature on the potential oscillation pulse signal, a quadratic polynomial regression model was established to describe the relationship between the component concentration, temperature and induction time: ln*t* = a_0_ + a_1_[KBrO_3_] + a_2_[MA] + a_3_[(NH_4_)_4_Ce(SO_4_)_4_] + a_4_T + a_5_[KBrO_3_][MA] + a_6_[KBrO_3_][Ce^4+^] + a_7_[KBrO_3_]T + a_8_[MA][Ce^4+^] + a_9_[MA]T + a_10_[Ce^4+^]T + a_11_[KBrO_3_]^2^ + a_12_[MA]^2^ + a_13_[Ce^4+^]^2^ + a_14_T^2^. Analysis of the predicted and measured values of the model’s fitted regression shows that the model has a high degree of significance and strong goodness of fit. The correlation coefficient is 0.998.
3. By studying the influence of the Cl^−^ concentration on the potential oscillation pulse signal, the results show that the relationship between the Cl^−^ concentration and induction time follows a secondary relationship described by the equation ln*t* = 5.337 − 0.775[Cl^−^] + 0.723[Cl^−^]^2^, T = 30 °C. The model demonstrated a high degree of fit, with a correlation coefficient of 0.987. An analysis of the predicted versus measured values confirms that the regression model is statistically significant and accurately represents the experimental data.
4. The results of the kinetic analysis and mechanism research on the influence of Cl^−^ on the induction time show that Cl^−^ does not participate in the electrochemical oscillation reaction but acts as an inhibitor of potential oscillation. As the concentration of Cl^−^ increases, it becomes more resistant to initiation, and the apparent activation energy increases. When the Cl^−^ concentration is less than 1 mmol/L, the induced apparent activation energy does not change much. As the Cl^−^ concentration increases from 1 mmol/L to 2 mmol/L, the induced apparent activation energy increases sharply from 50 kJ/mol to 120 kJ/mol. This significant increase severely hinders the reaction during the induction period of the B-Z oscillation reaction, greatly reduces the reaction speed, and then increases the induction time.

## 2. Results

### 2.1. Single-Factor Experiment with KBrO_3_

**Figure 1:**
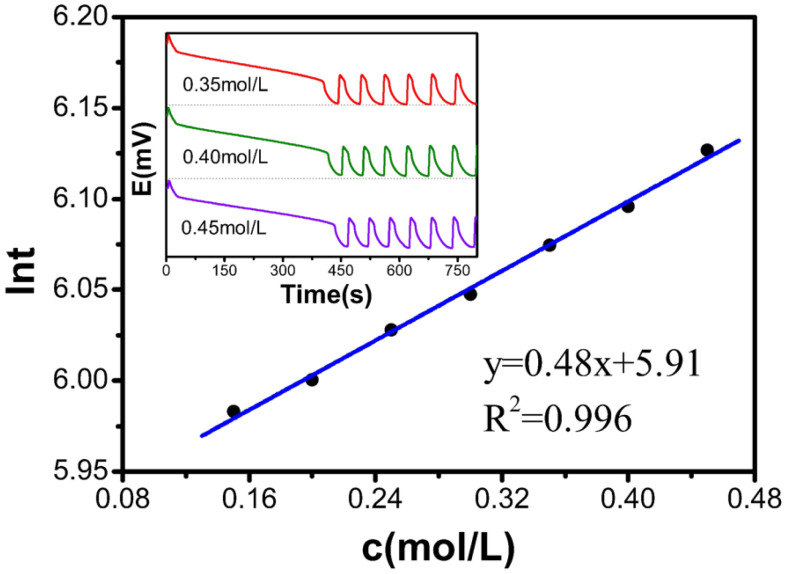
Influence of KBrO_3_ on induction time.

Table 1 and Figure 1 present that the c(KBrO_3_) increases from 0.15 mol/L to 0.45 mol/L, resulting in an increase in the induction time from 5.98 to 6.13. The relationship between the KBrO_3_ concentration and ln*t* follows a first-order linear equation: y = 0.48x + 5.91, with an R^2^ value of 0.996, indicating a good linear relationship. These results confirm that the induction time increases linearly with increasing KBrO_3_ concentration in the B-Z oscillating system.

### 2.2. Single-Factor Experiment with CH_2_(COOH)_2_

**Figure 2:**
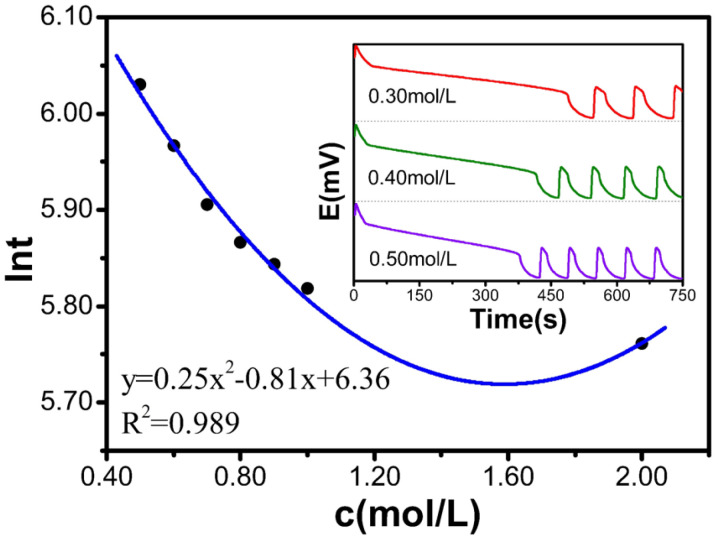
Influence of CH_2_(COOH)_2_ on the induction time.

Table 2 and Figure 2 display how c(CH_2_(COOH)_2_) affects the value of ln*t*. As c(CH_2_(COOH)_2_) increases from 0.50 mol/L to 2.00 mol/L, ln*t* decreases from 6.53 to 5.87. The relationship between the CH_2_(COOH)_2_ concentration and ln*t* satisfies the polynomial equation: y = 0.25x^2^ − 0.81x + 6.36, with a degree fit of 0.989, indicating a strong fit. Therefore, the experimental results show that the induction time decreases with increasing CH_2_(COOH)_2_ concentration.

**Figure 3:**
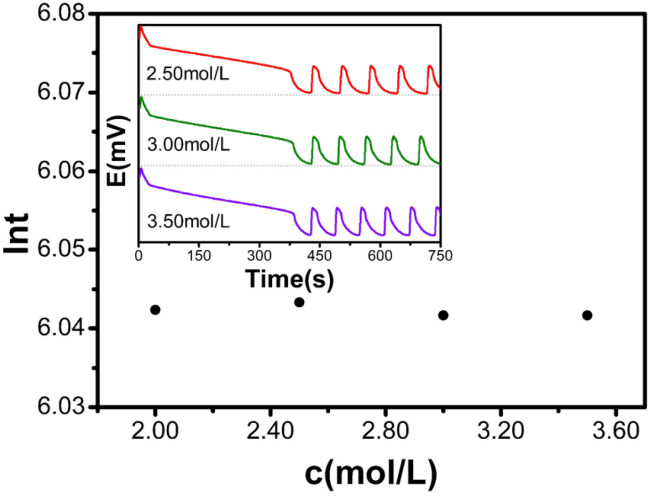
H_2_SO_4_ influence diagram on the induction time.

Table 3 and Figure 3 demonstrate that as the concentration of H_2_SO_4_ increases from 2.00 mol/L to 3.50 mol/L, the natural logarithm of the induction time remains virtually unchanged (approximately ln*t* ≈ 6.04). These results suggest that within this concentration range, H_2_SO_4_ has a negligible effect on the induction time of the B-Z oscillating system.

### 2.3. Single-Factor Experiment of (NH_4_)_4_Ce(SO_4_)_4_

**Figure 4:**
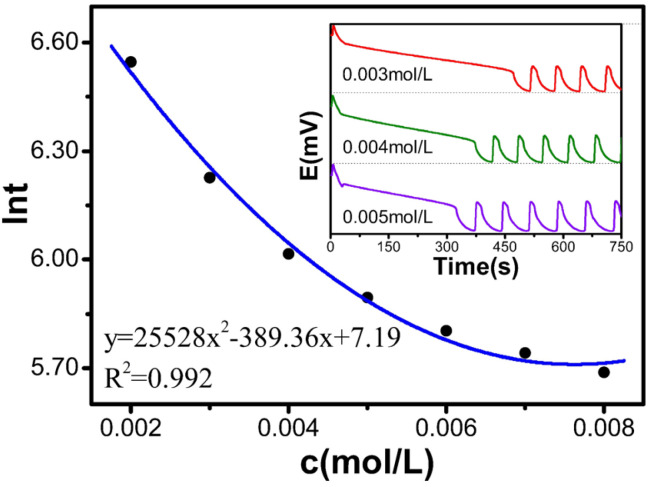
Influence diagram of (NH_4_)_4_Ce(SO_4_)_4_ on the induction time.

Table 4 and Figure 4 show that as the concentration of [(NH_4_)_4_Ce(SO_4_)_4_] increased from 0.002 to 0.008 mol/L, ln*t* decreased from 6.55 to 5.69. The relationship satisfies the polynomial equation: y = 25,528x^2^ − 389.36x + 7.19, with an R^2^ of 0.992. The experimental results indicate that the induction time decreases with increasing (NH_4_)_4_Ce(SO_4_)_4_ concentration.

### 2.4. Single-Factor Temperature Experiments

**Figure 5:**
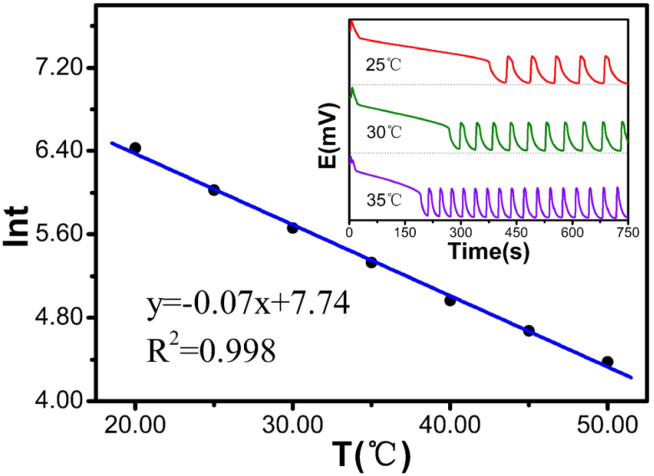
Temperature influence diagram of the induction time.

Table 5 and Figure 5 show that when the temperature increases from 20 °C to 50 °C, ln*t* decreases from 6.43 to 4.38. This relationship follows a linear equation: ln*t* is y = −0.07x + 7.74, with a degree of fit of 0.998, indicating a very strong linear correlation. The experimental results show that the induction time decreases significantly with increasing temperature.

### 2.5. Orthogonal Experiment to Optimize the Technological Conditions of the B-Z Oscillation System

Typically, the orthogonal experiment was carried out with 3.00 mol/L of H_2_SO_4_, and the other influencing factors were taken as independent variables, and the logarithm of the induction time was used as the evaluation index. The experimental results are shown in Table 6.

According to the data presented in Table 6, the logarithmic quadratic polynomial regression model of the induction time of the B-Z oscillation system was obtained through Design-Expert 8.0 software. The coefficient values of the log-quadratic polynomial model of induction time are shown in Table 7.

ln*t* = a_0_ + a_1_[KBrO_3_] + a_2_[MA] + a_3_[(NH_4_)_4_Ce(SO_4_)_4_] + a_4_T + a_5_[KBrO_3_][MA] + a_6_[KBrO_3_][Ce^4+^] + a_7_[KBrO_3_]T + a_8_[MA][Ce^4+^] + a_9_[MA]T + a_10_[Ce^4+^]T + a_11_[KBrO_3_]^2^ + a_12_[MA]^2^ + a_13_[Ce^4+^]^2^ + a_14_T^2^ (1)

**Figure 6:**
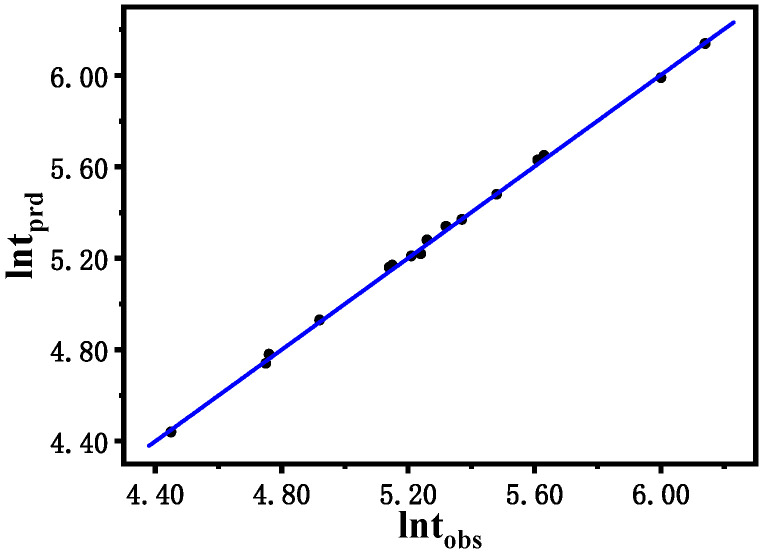
Relationship between the true value and the predicted value of ln*t*.

In Table 8, the *p* value can be used to judge the influence of the test factors on the logarithm of the induction time in order of temperature, cerium ammonium sulfate, malonic acid, and potassium bromate. The *p* value less than 0.0001 indicates that the regression model is highly significant and has a high degree of fit. *p* < 0.05 in the logarithmic regression model of induction time indicates that the regression term has a significant interaction effect in the regression model. The determination coefficient R^2^ and the adjusted determination coefficient of the model are both close to 1, and the coefficient of variation and precision are 0.61% and 61.923, respectively, which shows that the fitted regression model has high reliability. The relationship between the true values determined by the induction time and the predicted values of the fitted regression model is shown in Table 9 and Figure 6.

### 2.6. Optimization of the Process Conditions of the B-Z Oscillation System

The orthogonal test results show that the optimal experimental effect is when the [H_2_SO_4_] concentration is 3.00 mol/L, the [CH_2_(COOH)_2_] concentration is 1.10 mol/L, the [Ce^4+^] concentration is 0.007 mol/L, and the [KBrO_3_] concentration is 0.30 mol/L. Under these conditions, the system has the shortest induction time of 136.4 s. The oscillation system is relatively stable, and the periodicity of the oscillation curve is relatively good, with excellent reproducibility. Thus, the optimized program was used for subsequent research on how Cl^−^ influences the B-Z reaction.

**Figure 7:**
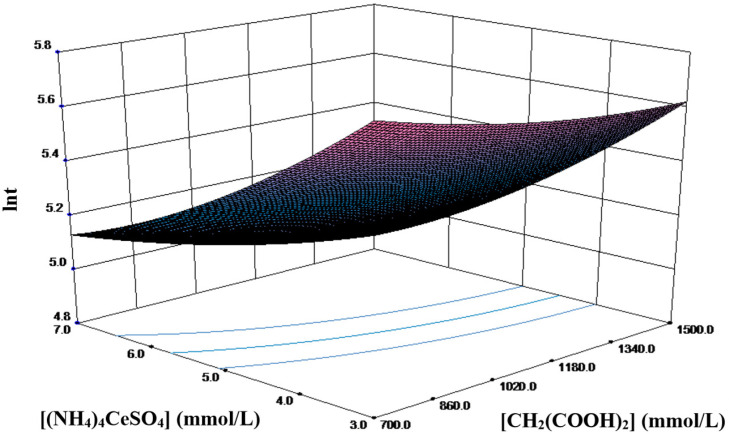
Response surface plot of the model for the B-Z reaction.

For a better representation of the response surface, only the parameters with the strongest interaction in the orthogonal experiment, namely, malonic acid and cerium amonium sulfate, were selected. Figure 7 shows the three-dimensional surface plot at the center level of the potassium bromate concentration ([KBrO_3_] = 0.35 mol L^−1^), which provides a graphical representation of the relationship between two independent factors ([CH_2_(COOH)_2_] and [(NH_4_)_4_Ce(SO_4_)_4_]) and ln*t*. The contour lines are slightly curved due to the twisting effect on the plane generated by the interaction of the parameters [CH_2_(COOH)_2_] and [(NH_4_)_4_Ce(SO_4_)_4_]. As expected from the sign of the coefficients, ln*t* increases with the increasing factor levels. Furthermore, the influence of the cerium ammonium sulfate concentration varies with the malonic acid concentration, suggesting a strong interaction between these two factors. The minimum ln*t* peak of 4.446 is observed when [CH_2_(COOH)_2_] = 1.50 mol L^−1^ and when [(NH_4_)_4_Ce(SO_4_)_4_] = 0.007 mol L^−1^.

### 2.7. Study of the Mechanism and Regulatory Effect of Cl^−^ on the B-Z Oscillation Reaction

The B-Z reaction system consisted of 10 mL of 1.10 mol/L malonic acid solution, 20 mL of 3.0 mol/L sulfuric acid solution, 20 mL of 0.30 mol/L potassium bromate solution, 20 mL of 0.007 mol/L cerium ammonium sulfate solution, and 10 mL of a certain concentration potassium chloride solution.

**Figure 8:**
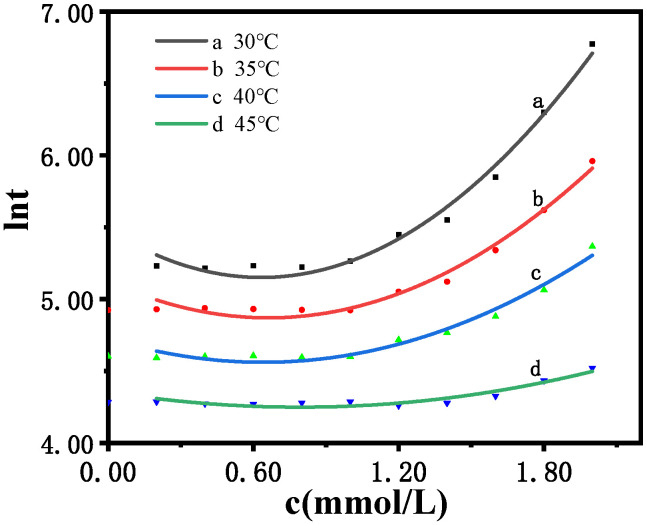
The logarithmic effect of the Cl^−^ concentration on the induction time of the B-Z oscillating reaction system at different temperatures.

Figure 8 and Table 10 show that, at 30 °C, as c(Cl^−^) increases from 0.20 mmol/L to 2.00 mmol/L, ln*t* increases from 5.23 to 6.77, and the concentrations of Cl^−^ and ln*t* satisfy the quadratic relationship described by Equation (2), with an R^2^ of 0.987, indicating an excellent fit. However, as the temperature increases, the reaction rate accelerates, and the induced reaction time becomes shorter, causing the quadratic relationship to gradually deteriorate. When it reaches 45 °C, the degree of fit is only 0.9135. Therefore, the experimental results show that the electrochemical oscillation based on the B-Z oscillation reaction generates a pulse signal. The addition of Cl^−^ to the system will affect certain elementary reactions in the system, which will interfere with the oscillation system. When the concentration of Cl^−^ ranged from 0.20 to 2.00 mmol/L, no evident change was detected for the initial induction time. However, as the Cl^−^ concentration reached 1 mmol/L, the oscillation induction time increased rapidly. As shown in Figure 8, the chemical information reflected by the changes in the shape of the oscillation curve can be used to determine the chlorine content.

At 30 °C, the parameter equation with three coefficients (a) was obtained via quadratic regression, as shown in Equation (2) and Table 11. The results indicate a quadratic dependence on [Cl^−^].

ln*t* = a_0_ + a_1_[Cl^−^] + a_2_[Cl^−^]^2^ (2)

**Figure 9:**
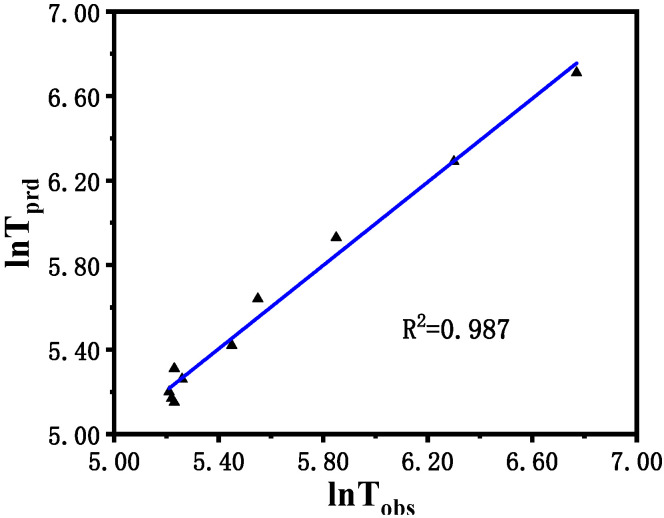
Predicted the logarithm of induction time (ln*t*_prd_) vs. observed the logarithm of induction time (ln*t*_obs_) of the B-Z reaction.

Figure 9 shows the comparison between the logarithm of the predicted induction time (ln*t*_prd_) calculated via the model described in (1) and the logarithm of the induction time (ln*t*_obs_) observed in the experiment. In general, the logarithmic value of the induction time ranges from 5.15 to 6.77, indicating a perfect agreement between the predicted value and the experimental result. The original residuals obtained from multiple regression are reasonably dispersed without heteroscedasticity and outliers.

According to the Arrhenius equation: lnk=−Ea/RT+C and $t^{-1}\propto k$, the following formula is derived: $lnt^{-1}=-Ea/RT+C_{1}$. The induced activation energy Ea can then be obtained via this formula according to the data in Table 10.

**Figure 10:**
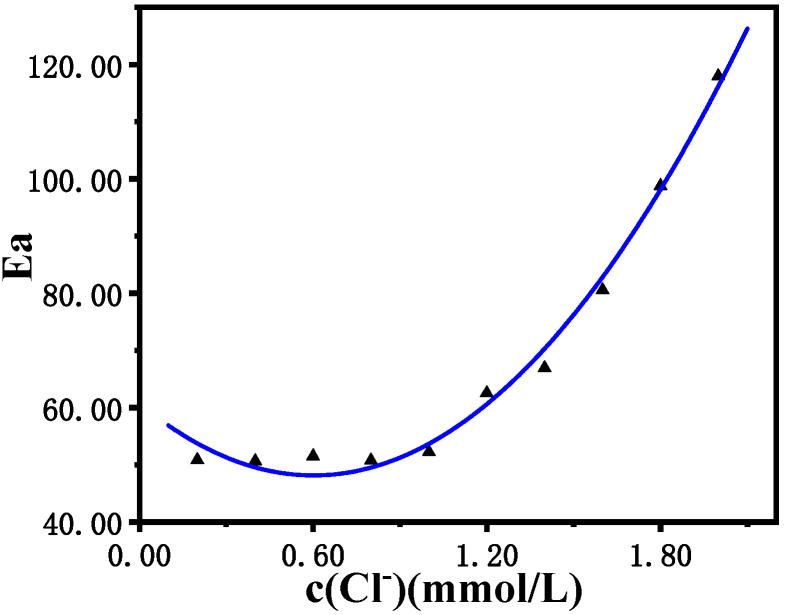
Relationship diagram of the influence of the Cl^−^ concentration on the induced activation energy Ea induced by the B-Z oscillating reaction system.

Figure 10 shows that with an increase in Cl^−^ concentration, the induced activation energy of the B-Z oscillation reaction system increases, and the induction reaction becomes more difficult to proceed. The fitting effect of the quadratic relationship is perfectly matched. When the concentration of Cl^−^ is lower than 1.0 × 10^−3^ mol/L, the induced activation energy (Ea) remains almost unchanged at 50 kJ/mol. However, as the concentration of Cl^−^ increases from 1.0 × 10^−3^ mol/L to 2.0 × 10^−3^ mol/L, the induced activation energy (Ea) increases rapidly from 50 kJ/mol to 120 kJ/mol, which severely hinders the reaction in the induction period of the B-Z oscillation reaction, and greatly reduces the reaction speed, increasing of the induction time.

**Figure 11:**
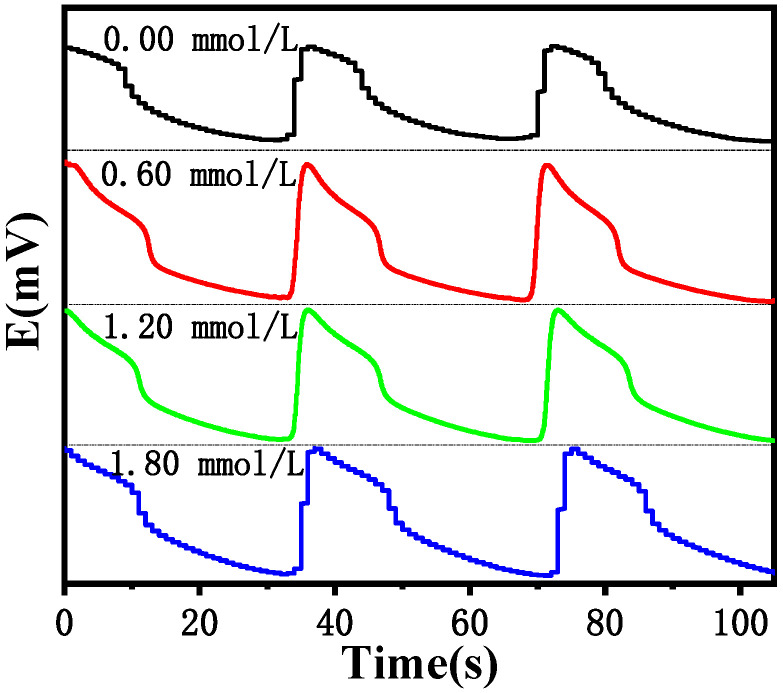
Influence of the oscillation period under different Cl^−^ concentration (Cl^−^) at 30 °C.

Figure 11 shows that the oscillation cycle is almost unchanged when different Cl^−^ concentrations are added. Therefore, the Cl^−^ does not affect the cycle time of the B-Z reaction, indicating that the Cl^−^ does not participate in the reaction, but is a strong inhibitor of the B-Z oscillation system, increasing the induction activation energy of the system and thereby affecting its induction time.

## 3. Materials and Methods

**Materials.** Potassium bromate (KBrO_3_), malonic acid (CH_2_(COOH)_2_), sulfuric acid (H_2_SO_4_), cerium ammonium sulfate ((NH_4_)_4_Ce(SO_4_)_4_), and potassium chloride (KCl) are analytical grade chemicals. Except for the preparation of the (NH_4_)_4_Ce(SO_4_)_4_ solution, 0.20 mol/L H_2_SO_4_ was used to prevent hydrolysis and turbidity. All other reagents were prepared with deionized water.

**Methods.** The chemical reaction followed the classic B-Z oscillatory reaction mechanism. The constant-temperature water was passed through the reactor jacket, and the super constant temperature bath was turned on to adjust the water temperature. In our experiments, we utilized a CHI660E electrochemical workstation, which is equipped with a standard three-electrode system. The working electrode was a glassy carbon electrode, the counter electrode was a platinum wire, and the reference electrode was the Ag/AgCl electrode. Data acquisition and analysis were performed via the software provided by CHI. The CH_2_(COOH)_2_ solution, KBrO_3_ solution, and H_2_SO_4_ solution were thoroughly mixed in a jacket reactor, and the preheated (NH_4_)_4_Ce(SO_4_)_4_ solution was added after 10 min at a constant temperature to determine the electrochemical oscillation curve. The reaction was carried out under stirring at 600 r/min. In this study, each experiment was conducted three times to ensure the reproducibility of the results.

## 4. Conclusions

In this work, a mathematical model was constructed via both single-factor experiments and multivariate statistical analysis, which illustrates that the temperature and cerium ammonium sulfate concentration are the most influential parameters affecting the induction time. As the temperature increases and the concentration of cerium ammonium sulfate increases, the induction time decreases accordingly. As the temperature increased, the induction time decreased as the concentration of cerium ammonium sulfate increased. Orthogonal experiments on the four factors of potassium bromate, malonic acid, cerium ammonium sulfate, and temperature were used to determine the optimal reaction conditions for the B-Z oscillation with a shorter induction time and better periodicity.

Afterward, on the basis of an optimized system determined by the above orthogonal experiment, we studied the influence of different concentrations of Cl^−^ on the induction time of the system. The results show that there is a good quadratic relationship between the Cl^−^ concentration and the induction time of the system, and the logarithmic value of the induction time predicted by the relationship model is in good agreement with the experimental results. A comparison of the B-Z oscillation periods at different Cl^−^ concentrations revealed that Cl^−^ does not affect the period of the B-Z reaction, indicating that Cl^−^ does not participate in the reaction. However, Cl^−^ strongly inhibits the B-Z oscillation system by increasing the induction activation energy and thereby affecting the induction time.

## Figures and Tables

**Table 1:** Relationship between the concentration of the KBrO_3_ solution and the reaction.

c[M]	t(s)	ln*t*
0.15	396.6	5.983
0.20	403.6	6.000
0.25	414.8	6.028
0.30	423.0	6.047
0.35	434.7	6.075
0.40	444.0	6.096
0.45	458.0	6.127

**Table 2:** Relationship between the concentration of the CH_2_(COOH)_2_ solution and the reaction.

c[M]	t(s)	ln*t*
0.50	415.9	6.030
0.60	390.2	5.967
0.70	367.1	5.906
0.80	352.9	5.866
0.90	345.1	5.844
1.00	336.4	5.818
2.00	317.7	5.761

**Table 3:** Relationship between the concentration of H_2_SO_4_ solution and induction time in the reaction.

c[M]	t(s)	ln*t*
2.00	420.9	6.042
2.50	421.3	6.043
3.00	420.6	6.042
3.50	420.6	6.042

**Table 4:** Relationship between the concentration of the catalyst solution and the reaction.

c[M]	t(s)	ln*t*
0.002	697.2	6.547
0.003	505.6	6.226
0.004	409.6	6.015
0.005	363.1	5.895
0.006	331.6	5.804
0.007	311.5	5.741
0.008	295.6	5.689

**Table 5:** Relationship between temperature and the reaction.

T(°C)	t(s)	ln*t*
20.00	618.1	6.427
25.00	413.4	6.024
30.00	287.5	5.661
35.00	207.2	5.334
40.00	143.8	4.968
45.00	107.0	4.673
50.00	79.5	4.376

**Table 6:** Length of induction time measured for 21 experiments established in a design with a center point for the B-Z reaction. In this experiment, [H_2_SO_4_] = 3 mol L^−1^. The factors are (A)[KBrO_3_]/mol L^−1^, (B)[CH_2_(COOH)_2_]/mol L^−1^, (C) [(NH_4_)_4_Ce(SO_4_)_4_]/mol L^−1^, (D)T/°C.

Experiment	A	B	C	D	ln*t*
1	0.30	1.10	0.005	30.00	5.237
2	0.30	1.10	0.005	30.00	5.244
3	0.35	1.10	0.005	30.00	5.258
4	0.30	1.10	0.007	30.00	4.916
5	0.30	1.10	0.005	35.00	4.764
6	0.30	1.50	0.005	30.00	5.152
7	0.30	1.10	0.005	30.00	5.243
8	0.35	0.70	0.007	35.00	4.746
9	0.25	1.50	0.003	35.00	5.212
10	0.25	0.70	0.003	25.00	6.144
11	0.35	0.70	0.003	35.00	5.373
12	0.25	1.50	0.007	35.00	4.446
13	0.30	1.10	0.005	30.00	5.237
14	0.25	1.10	0.005	30.00	5.136
15	0.35	1.50	0.003	25.00	5.997
16	0.25	0.70	0.007	25.00	5.479
17	0.30	1.10	0.003	30.00	5.608
18	0.30	1.10	0.005	25.00	5.628
19	0.35	1.50	0.007	25.00	5.213
20	0.30	1.10	0.005	30.00	5.236
21	0.30	0.70	0.005	30.00	5.319

**Table 7:** Coefficient values of the log-quadratic polynomial model of induction time.

Constant	Value	Constant	Value	Constant	Value
a_0_	10.2	a_5_/L^2^ mol^−2^	−2.5	a_10_/L mol^−1^ °C^−1^	0.68
a_1_/L mol^−1^	0.52	a_6_/L^2^ mol^−2^	23.29	a_11_/L^2^ mol^−2^	0.45
a_2_/L mol^−1^	−0.62	a_7_/L mol^−1^ °C^−1^	0.1	a_12_/L^2^ mol^−2^	0.25
a_3_/L mol^−1^	−323.53	a_8_/L^2^ mol^−2^	−40.34	a_13_/L^2^ mol^−2^	16,382.072
a_4_/°C	−0.15	a_9_/L mol^−1^ °C^−1^	0.03	a_14_/°C^−2^	−18.4

**Table 8:** Analysis of variance of the log-quadratic polynomial model of induction time. “**” means this item is extremely significant (*p* < 0.01); “*” means this item is significant (*p* < 0.05).

Source of Variance	Mean Square	Degree of Freedom	Sum of Square	*p* Value
Model	0.21	14	2.96	<0.0001 **
[KBrO_3_]	7.397 × 10^−3^	1	7.397 × 10^−3^	0.0376 *
[MA]	0.014	1	0.014	0.0106 *
[Ce^4+^]	1.25	1	1.25	<0.0001 **
T	0.37	1	0.37	<0.0001 **
[KBrO_3_] [MA]	4.004 × 10^−3^	1	4.004 × 10^−3^	0.0984
[KBrO_3_] [Ce^4+^]	4.338 × 10^−5^	1	4.338 × 10^−5^	0.8455
[KBrO_3_] T	1.042 × 10^−3^	1	1.042 × 10^−3^	0.3571
[MA] [Ce^4+^]	8.334 × 10^−3^	1	8.334 × 10^−3^	0.0303 *
[MA] T	4.821 × 10^−3^	1	4.821 × 10^−3^	0.0756
[Ce^4+^] T	3.703 × 10^−4^	1	3.703 × 10^−4^	0.5738
[KBrO_3_]^2^	3.170 × 10^−6^	1	3.170 × 10^−6^	0.9576
[MA]^2^	3.931 × 10^−3^	1	3.931 × 10^−3^	0.1008
[Ce^4+^]^2^	0.011	1	0.011	0.0178 *
T^2^	2.872 × 10^−7^	1	2.872 × 10^−7^	0.9873
Residual	1.047 × 10^−3^	6	6.284 × 10^−3^	
Pure error	1.511 × 10^−5^	4	6.044 × 10^−5^	
Sum		20	2.97	

**Table 9:** Comparison of the true values and predicted values.

Actual Value	Predicted Value
5.21	5.21
6.00	5.99
4.75	4.74
5.21	5.21
5.37	5.37
5.48	5.48
4.45	4.44
6.14	6.14
5.14	5.16
5.26	5.28
5.32	5.34
5.15	5.17
5.61	5.63
4.92	4.93
5.63	5.65
4.76	4.78
5.24	5.22
5.24	5.22
5.24	5.22
5.24	5.22
5.24	5.22

**Table 10:** Influence of the Cl^−^ solution concentration on the reaction at different temperatures.

Concentration (mmol)	Induction Time (s)			
	30 °C	35 °C	40 °C	45 °C
0.00	5.23	4.92	4.60	4.28
0.20	5.23	4.93	4.59	4.29
0.40	5.21	4.94	4.60	4.27
0.60	5.23	4.93	4.61	4.27
0.80	5.22	4.93	4.59	4.28
1.00	5.26	4.92	4.60	4.29
1.20	5.45	5.05	4.72	4.26
1.40	5.55	5.12	4.77	4.28
1.60	5.85	5.34	4.88	4.33
1.80	6.30	5.62	5.06	4.43
2.00	6.77	5.96	5.37	4.52

**Table 11:** Coefficient values of the fitting relationship induced by Cl^−^ at 30 °C.

Constant	Value
a_0_	5.337
a_1_/L mol^−1^	−0.775
a_2_/L^2^ mol^−2^	0.723

## Data Availability

The original contributions presented in this study are included in the article. Further inquiries can be directed to the corresponding author.
